# Penetrating thoracic ice pick injury extending into pulmonary artery: Report of a case

**DOI:** 10.1016/j.ijscr.2018.09.052

**Published:** 2018-10-08

**Authors:** Marina Kawaguchi, Hirotsugu Yamamoto, Taihei Yamada, Tetsuya Yumoto, Toshiyuki Aokage, Hiromi Ihoriya, Koki Eto, Takanori Suezawa, Hiromichi Naito, Atsunori Nakao

**Affiliations:** a Department of Emergency, Critical Care and Disaster Medicine, Okayama University Graduate School of Medicine, Dentistry and Pharmaceutical Sciences, Japan; b Department of Cardiovascular Surgery, Okayama University Graduate School of Medicine, Dentistry and Pharmaceutical Sciences, Japan

**Keywords:** Penetrating chest injury, Pulmonary artery, Emergency surgery, Case report

## Abstract

**Introduction:**

Penetrating thoracic traumas have a wide spectrum, ranging from mild traumas to life-threatening injuries.

**Presentation of case:**

This paper illustrates a 40-year-old male with a penetrating pulmonary artery injury that was successfully treated with emergency surgery. The patient visited local hospital by foot complaining of moving object on his chest and was found that an ice pick was penetrating the man's left chest. An ambulance took the patient to our emergency department. Computed tomography of the chest showed linear metallic density in the pulmonary trunk and a small amount of pericardial fluid. Emergency surgery for removal of the object and repair of the pulmonary artery was performed. The ice pick had been stuck in the main pulmonary artery through the pericardium without any injury to the left lung or internal thoracic artery. Postoperative course was uneventful.

**Discussion:**

A multidisciplinary team designed our treatment strategy, enabling us to select the optimal treatment, including diagnostic techniques and surgical approach. A successful trauma management depends on whether a life-threatening conditionexists. Major vascular injury should be rapidly assessed in these cases. Psychiatric aspects of the case are also discussed.

**Conclusion:**

Early removal of the foreign body is recommended to prevent further damage to the heart.

## Highlights

• A chest stab wound can have various clinical presentations ranging from no intrathoracic injury to life-threatening, extensive damage to the great vessels.
• Early removal of the foreign body is recommended to prevent further damage to the heart.
• Emergency physicians should seriously consider psychiatric consultation to prevent repeated suicidal attempts.

## 1 Introduction

Penetrating thoracic injury is a life-threatening trauma with a high incidence of death from hemorrhagic shock or pericardial tamponade before the patient arrives at the hospital [[1], [2], [3]]. We present a 40-year-old male with a penetrating pulmonary artery injury that was successfully treated with emergency surgery.

A multidisciplinary team designed our treatment strategy, enabling us to select the optimal treatment, including diagnostic techniques and surgical approach. The psychological aspects of this case were challenging, as the patient denied the injury was self-inflicted and no criminal activity associated with the case was found upon police investigation. Our experience may help emergency physicians treat such a case. Although rare, this condition can be associated with significant morbidity. This case report was reported in line with the SCARE criteria [4].

## 2 Case presentation

**Fig. 1:**
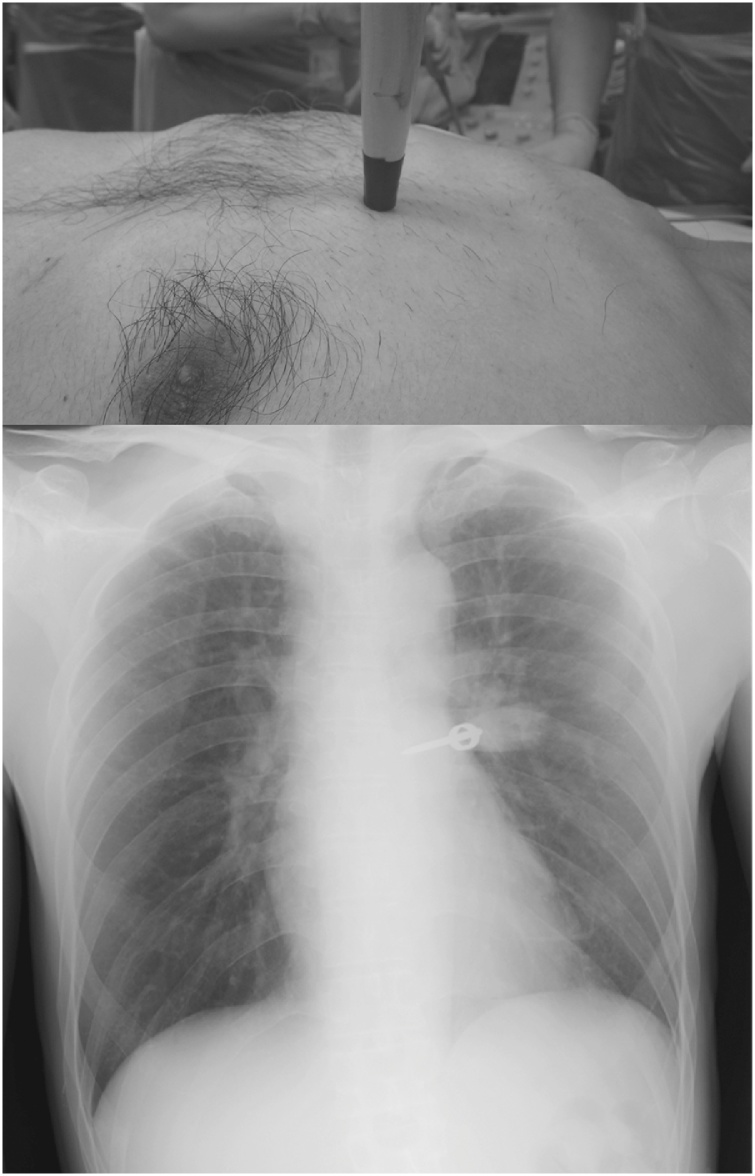
The site and injury pattern of the stabbing (Upper panel). Chest X-ray revealed a clear lung field without pneumothorax (Lower panel).

A 40-year-old man was taken to the local hospital by his office supervisor by foot due to concerns over moving a foreign object impaling his chest (Fig. 1A). As soon as the emergency physician noted that an ice pick was penetrating the man’s left chest, we established an intravenous line while preventing the object from moving from its original position, and an ambulance took the patient to our emergency department.

On examination, the patient’s general condition was not critical (Glasgow Coma Scale score 15, respiratory rate 24 breaths/min, blood pressure 123/79 mmHg, heart rate 76 beats/min, oxygen saturation 100% on 2 L nasal cannula, and body temperature 37.2 °C). The patient would not reveal the actual cause of the injury. An interview with his family disclosed no history of depressive disorder, pharmacological treatment, substance use, or alcohol abuse, but the patient had a history of several pneumothorax injuries, one 10 years prior and two a year prior to this episode. In all episodes, he had been hospitalized for several days for chest drainage and discharged without any complications. Considering his condition, we strongly suspected a self-inflicted injury. However, our patient denied any suicidal ideation, depressive mood, or hopelessness. His family members described him as a quiet and gentle person who did not behave impulsively.

**Fig. 2:**
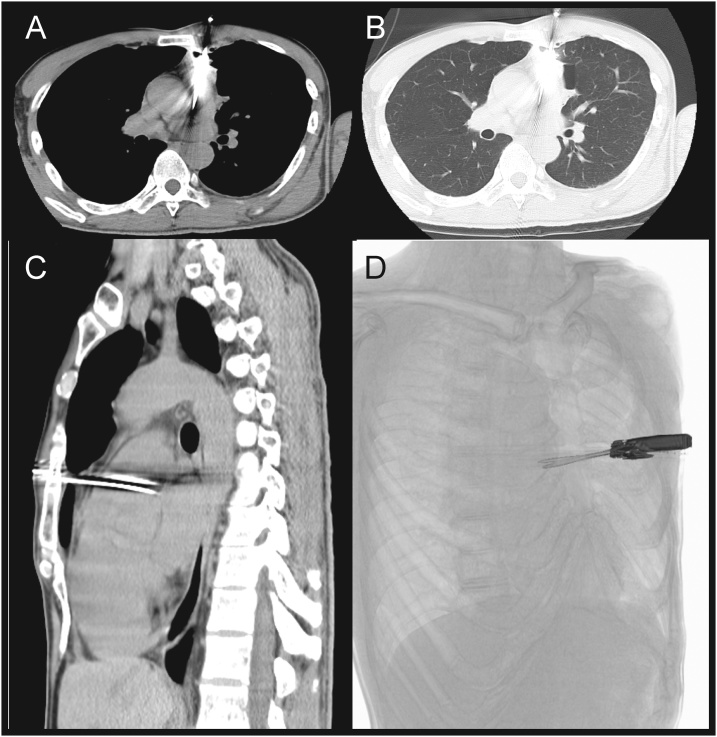
Computed tomography of the chest showed linear metallic density in the pulmonary trunk and a small amount of pericardial fluid.

Cardiovascular auscultation was unremarkable without murmurs or gallops. Chest X ray demonstrated a clear lung field without pneumothorax or hemothorax (Fig. 1B). Emergency echocardiography disclosed a small amount of pericardial effusion without cardiac tamponade. Computed tomography (CT) of the chest showed linear metallic density in the pulmonary trunk and a small amount of pericardial fluid (Fig. 2A–D). Pneumothorax or bulla was not seen on chest CT. Based on the diagnosis of penetrating cardiac injury, we transferred the patient to the operating theater after cardiac surgery consultation.

We placed the patient in the supine position. After performing a full median sternotomy, the pericardium was opened. There was a small amount of pericardial effusion that seemed to be mixed with blood. The ice pick had been stuck in the main pulmonary artery (MPA) through the pericardium without any injury to the left lung or internal thoracic artery. Heparin was given and cardiopulmonary bypass (CPB) was commenced with ascending aortic and bicaval cannulation. The patient was cooled down to 32 ° Celsius. After aortic cross clamping, the cardioplegia was infused into the aortic root to obtain cardiac arrest. A longitudinal incision was made in the MPA, and we carefully removed the foreign body, which was lodged through the MPA from the anterior to posterior wall near the annulus of the pulmonary valve without injury to the left main coronary trunk. The holes made by the ice pick were closed using 5-0 polypropylene suture. The MPA was closed using 4-0 polypropylene over and over running suture. After rewarming and deairing, the aortic clamp was removed. The patient’s sinus rhythm came back spontaneously. CPB weaning was smooth, and protamine was given. The chest was closed in a normal fashion. Postoperative recovery was uneventful.

A police investigation showed no criminal events associated with the injury. Although the patient denied stabbing himself with the ice pick, we strongly suspected the penetrating cardiac injury was self-inflicted and consulted the psychiatric department on day 7 to closely monitor the patient during hospitalization. During the psychiatric counseling sessions after the surgery, the patient continued to stubbornly deny a suicide attempt. At 16 days POD, the patient was discharged to home without psychiatric follow-up.

## 3 Discussion

Emergency clinicians should always approach penetrating thoracic injuries with the utmost caution and use advanced diagnostics as soon as possible to confirm or rule out vital organ damage. In cases of possible or definite organ injury, fluid support should be given at once and the patient should be moved to the operating room immediately [5,6]. When we first encountered our patient, the ice pick had not been moved from its original position, and the patient was quickly examined and taken to the operating room.

The general rules of managing penetrating trauma are to avoid in-depth exploration when assessing the wound site, refrain from removing the penetrating object before an accurate diagnosis is known, and be prepared to possibility intubate for airway security at any moment [7]. Surgical management could be limited to extracting the ice pick in the operating room. We completely followed these therapeutic strategies. The applied multispecialist approach resulted in a successful outcome.

Most patient deaths from penetrating chest trauma are due to serious vascular injury. Our patient was lucky enough in that there was no evidence of injury to the left main coronary trunk, which is located behind the annulus of the pulmonary valve. Also, since the injury was mainly seen in the right-heart system with low pressure, critical complications including cardiac tamponade did not occur [8]. Importantly, the psychological aspects of our case were quite challenging. Considering that this patient had previously undergone multiple chest tube insertions for recurrent spontaneous pneumothoraxes, we may assume that he previously impulsively attempted suicide by stabbing himself in the chest. A factitious disorder may have produced the physical symptoms of self-injury. The patient should have remained hospitalized until completion of the treatment, but the patient consented to leave the hospital after 8 days of psychological counseling, despite the medical team’s advice. Unfortunately, our patient refused psychiatric follow-up after discharge, preventing us from further studying this unusual case. Although at this moment we have not encountered another unusual episode with this patient, we fear that he may stab himself in the chest again in the near future. We should be aware that psychiatric problems are often underdiagnosed.

## 4 Conclusion

A chest stab wound can have various clinical presentations ranging from no intrathoracic injury to life-threatening, extensive damage to the great vessels. Early removal of the foreign body is recommended to prevent further damage to the heart. Importantly, emergency physicians should seriously consider psychiatric consultation to prevent repeated suicidal attempts.

## Conflict of interest

All authors of this manuscript declare no conflicts of interests.

## Sources of funding

No funding support was given for this study.

## Ethical approval

This study was approved by Okayama University Hospital Ethical Committee.

## Consent

Written informed consent was obtained from the patient for publication of this case report and accompanying images. A copy of the written consent is available for review by the Editor-in-Chief of this journal on request”.

## Author contribution

Marina Kawaguchi, Hirotsugu Yamamoto, Taihei Yamada, Tetsuya Yumoto, Shigeyuki Aokage, Hiromi Ihoriya, and Hiromichi Naito contributed to the study design, data collections, data analysis, writing and review. Koki Eto, Takanori Suezawa performed surgery. Atsunori Nakao contributed to the data collections and review.

## Registration of research studies

Not applicable.

## Guarantor

Atsunori Nakao, MD.

## Provenance and peer review

Not commissioned, externally peer reviewed.
